# A Comprehensive Review of Current Trends in the Diagnosis and Treatment of Ovarian Germ Cell Tumors

**DOI:** 10.7759/cureus.52650

**Published:** 2024-01-21

**Authors:** Ketki S Dantkale, Manjusha Agrawal

**Affiliations:** 1 Department of Obstetrics and Gynecology, Jawaharlal Nehru Medical College, Datta Meghe Institute of Higher Education & Research (DMIHER), Wardha, IND

**Keywords:** multidisciplinary care, prognosis, treatment modalities, diagnosis, histological subtypes, ovarian germ cell tumors

## Abstract

Ovarian germ cell tumors constitute a rare and intricate spectrum of neoplasms characterized by diverse histological subtypes. This comprehensive review elucidates the classification, diagnosis, treatment, prognosis, and unique challenges associated with these tumors. The classification is rooted in histological attributes, with principal subtypes encompassing dysgerminoma, immature teratoma, yolk sac tumor (endodermal sinus tumor), choriocarcinoma, and mixed germ cell tumors. Each subtype bears distinct characteristics and clinical implications, necessitating precise diagnosis and tailored therapeutic strategies. Diagnosis hinges upon recognizing the broad clinical presentation, employing imaging techniques (such as ultrasound and MRI), evaluating tumor markers (alpha-fetoprotein and beta-human chorionic gonadotropin), and conducting histopathological examinations where necessary. Staging, primarily utilizing the International Federation of Gynecology and Obstetrics (FIGO) system, is pivotal in determining the extent of disease, guiding treatment choices, and facilitating prognostic assessment. Treatment modalities encompass surgery, chemotherapy (including standard regimens and emerging therapies), radiation therapy, targeted therapies, and immunotherapy. Prognosis is influenced by histological subtype, tumor stage, patient age, surgical success, response to chemotherapy, and tumor markers, while predictive biomarkers are continually emerging. Despite advances in treatment, ovarian germ cell tumors pose distinct challenges, including late diagnosis, treatment-related side effects, and the enigma of chemoresistance. An integral aspect of comprehensive care is supportive strategies to manage symptoms and offer psychological and emotional support. This review accentuates the vital role of early diagnosis and multidisciplinary care in optimizing outcomes. Future research directions and evolving clinical practices are explored in these intricate and distinctive malignancies, highlighting the dynamic landscape of ovarian germ cell tumors.

## Introduction and background

Ovarian germ cell tumors are a rare neoplasm originating from the ovaries' germ cells. Unlike epithelial ovarian cancer, which is more common in adult women, germ cell tumors primarily affect younger individuals, often occurring in the first three decades of life. These diverse tumors have various histological subtypes, including dysgerminomas, immature teratomas, yolk sac tumors, choriocarcinomas, and mixed germ cell tumors. Their histological diversity and unique characteristics present distinct challenges in diagnosis and treatment [1].

Early diagnosis and prompt, effective treatment is paramount in managing ovarian germ cell tumors. Due to their occurrence in younger patients and their potential to proliferate, early detection can significantly impact prognosis and quality of life. Delays in diagnosis may result in the tumors reaching an advanced stage, limiting treatment options and overall survival. Therefore, understanding the latest trends and advancements in diagnosing and treating these tumors is crucial to improving patient outcomes [2].

This comprehensive review aims to provide an in-depth examination of the current trends in the diagnosis and treatment of ovarian germ cell tumors. This review will encompass the latest research, clinical guidelines, and emerging therapies of the field. By synthesizing and analyzing this information, we aim to provide healthcare professionals, researchers, and the medical community with a comprehensive resource to enhance their understanding of ovarian germ cell tumors.

## Review

Classification and epidemiology

Classification of Ovarian Germ Cell Tumors

Dysgerminoma: Dysgerminomas represent the most common subtype of ovarian germ cell tumors and are predominantly found in adolescents and young adults. These tumors are characterized by a uniform population of large cells featuring clear cytoplasm. The distinct uniformity in cell type and clear cytoplasm sets them apart histologically. Dysgerminomas are often associated with favorable outcomes, being highly sensitive to chemotherapy and radiation therapy, making them an essential focus for early diagnosis and treatment [3].

Immature teratoma: Immature teratomas display a unique blend of differentiated and undifferentiated tissues, resembling embryonic development. These tumors tend to be diagnosed in younger patients. They are typically composed of a wide variety of tissue types, reflecting the potential of germ cells to differentiate into diverse structures. Prognosis in cases of immature teratomas is influenced by the degree of differentiation, with more undifferentiated components potentially indicating a less favorable outcome. Timely and comprehensive surgical resection is often the primary treatment approach [4].

Yolk sac tumor (endodermal sinus tumor): Yolk sac tumors are frequently observed in children and young women. They typically present a microcystic or reticular pattern associated with elevated alpha-fetoprotein (AFP) levels in the bloodstream. This unique marker is a critical diagnostic tool and monitoring parameter for yolk sac tumors. Many patients can achieve positive outcomes with prompt diagnosis and appropriate treatment, including chemotherapy, although successful management is essential given their potential aggressiveness [5].

Choriocarcinoma: Choriocarcinomas are rare and set themselves apart by the presence of trophoblastic tissue. These tumors may produce substantial amounts of beta-human chorionic gonadotropin (β-hCG), another critical tumor marker. Choriocarcinomas are highly aggressive, and early diagnosis and aggressive treatment are imperative. Their rarity and aggressive nature necessitate specialized care and swift intervention to optimize outcomes [6].

Mixed germ cell tumors: Mixed germ cell tumors are a complex category composed of two or more germ cell elements, such as combinations of dysgerminoma and yolk sac tumor. The prognosis for patients with mixed germ cell tumors can vary significantly, contingent on the histological components involved. Treatment approaches must be precisely tailored based on the specific histology and the tumor stage, emphasizing the importance of personalized care [7].

Incidence and Prevalence

Ovarian germ cell tumors are relatively rare compared to epithelial ovarian tumors. Their incidence varies across different regions but they predominantly affect young women. The exact prevalence depends on factors such as geography and population demographics. Malignant ovarian germ cell tumors (MOGTs) are rare, accounting for only 3-5% of ovarian cancers. Additionally, the incidence of germ cell tumors varies greatly depending on age. The incidence of MOGTs rises steadily in young girls from age nine to late teens between ages 15 and 19. While germ cell tumors represent a small fraction of all ovarian tumors, their unique characteristics and challenges make them an essential focus of research and clinical attention [8].

Age Distribution and Risk Factors

Ovarian germ cell tumors exhibit a distinct age distribution compared to other ovarian cancers. Most cases occur in young women, with a peak incidence in the second and third decades of life. Risk factors for ovarian germ cell tumors are not as well-defined as other ovarian malignancies. However, specific genetic syndromes such as Swyer syndrome (46, XY pure gonadal dysgenesis) may be associated with an increased risk of these tumors. Research into genetic predispositions and environmental factors contributing to the development of these tumors is ongoing [9].

Diagnosis

Clinical Presentation and Symptoms

Diagnosing ovarian germ cell tumors begins with a comprehensive clinical presentation and symptoms assessment. Ovarian germ cell tumors can present with a spectrum of signs and symptoms, underscoring the heterogeneity of their clinical manifestation. These symptoms may encompass abdominal pain or discomfort, which can range in intensity from mild to severe, and abdominal swelling or bloating due to tumor growth. Notably, younger patients might experience menstrual irregularities including changes in their menstrual cycles such as irregular periods or amenorrhea. Pelvic pain, another common symptom, can contribute to the clinical picture. In some cases, complications such as ovarian torsion or rupture may occur resulting in acute abdominal pain, making it critical to consider these tumors as a potential cause of such emergencies. Additionally, during physical examinations, healthcare providers may detect palpable masses or lumps in the pelvic or abdominal regions [10].

The diverse clinical presentation of ovarian germ cell tumors can sometimes be nonspecific, posing diagnostic challenges. Hence, maintaining a high index of suspicion, especially in young women with unexplained abdominal symptoms, is paramount to ensuring early diagnosis and timely intervention. Raising awareness among patients and healthcare providers about these symptoms and the potential presence of ovarian germ cell tumors is crucial in facilitating early detection and improving patient outcomes [11].

Imaging Techniques

Ultrasound: Transvaginal ultrasound is an invaluable initial evaluation tool for ovarian tumors. This non-invasive imaging technique provides essential information about the tumor's characteristics and relation to surrounding structures. A small probe is inserted into the vagina during a transvaginal ultrasound, allowing for a closer examination of the pelvic area. It can effectively identify the presence of an ovarian mass; assess its size, location, and characteristics, and distinguish between solid and cystic components. Moreover, Doppler ultrasound, which assesses blood flow within the tumor, can offer insights into the tumor's vascularity. This information is critical in the early stages of diagnosis and can guide decisions about the need for further evaluation and intervention [12].

MRI: MRI is a highly sensitive and detailed imaging modality that plays a pivotal role in the comprehensive evaluation and staging of ovarian germ cell tumors. MRI provides information about the tumor's size, location, characteristics, and relation to adjacent structures within the pelvis and abdomen. This level of detail is beneficial for preoperative evaluation, surgical planning, and staging. MRI is instrumental in identifying the extent of the tumor, assessing its potential involvement with nearby organs, and detecting the presence of any lymph node enlargement or metastasis. The ability to visualize these features allows for more accurate staging, which helps healthcare providers tailor treatment strategies to the individual patient's condition [13].

Tumor Markers

AFP: Elevated levels of AFP are often a hallmark of specific subtypes of ovarian germ cell tumors, primarily yolk sac tumors. While it is most associated with yolk sac tumors, AFP elevation can occur in rare instances with other germ cell tumor subtypes. AFP serves a dual role in the management of these tumors. First, it plays a vital role in the initial diagnosis as significantly elevated AFP levels in the blood can strongly suggest the presence of a germ cell tumor, prompting further evaluation and diagnostic procedures. Second, AFP is a crucial marker for monitoring treatment response. During therapy, a decrease in AFP levels indicates a positive response to treatment, while persistent or rising levels may signal resistance or disease progression. Regular monitoring of AFP allows healthcare providers to adapt treatment strategies to optimize the patient's chances of a successful outcome [14].

β-hCG: Choriocarcinomas and some mixed germ cell tumors have the potential to produce β-hCG, making it a valuable tumor marker for these specific subtypes. Elevated β-hCG levels indicate the presence of choriocarcinoma or mixed germ cell tumors. This marker is not only essential for diagnosis but also for the ongoing management of these tumors. Healthcare providers monitor β-hCG levels during treatment to assess the patient's response. A decline in β-hCG levels indicates a favorable response to therapy, while persistent elevations may suggest treatment resistance or disease progression. As with AFP, regular monitoring of β-hCG levels guides treatment decisions and helps optimize the patient's care [15].

Histopathological Examination and Differential Diagnosis

The definitive diagnosis of ovarian germ cell tumors is based on histopathological examination of the tumor tissue obtained through surgical procedures such as biopsy or surgical resection. Histopathology allows for identifying the specific germ cell tumor subtype, assessing tumor grade, and determining associated components such as teratomatous elements [7]. Ovarian germ cell tumors share clinical features with other ovarian neoplasms and conditions including epithelial ovarian tumors, sex cord-stromal tumors, and benign cysts. Differential diagnosis is essential to distinguish between these entities. Detailed histopathological examination and clinical, imaging, and laboratory findings help differentiate ovarian germ cell tumors from other ovarian masses [16].

Staging

FIGO Staging System

Staging ovarian germ cell tumors is crucial for determining the extent of the disease and guiding treatment decisions. The International Federation of Gynecology and Obstetrics (FIGO) staging system, commonly used for ovarian cancer, is also applied to ovarian germ cell tumors. The stages in the FIGO staging system for ovarian germ cell tumors are defined in Table 1 [17].

**Table 1:** Outlining the FIGO staging system for ovarian germ cell tumors FIGO: International Federation of Gynecology and Obstetrics

Stage	Description
Stage I	The tumor is confined to one or both ovaries.
Stage II	The tumor involves one or both ovaries with pelvic extension.
Stage III	The tumor involves one or both ovaries with microscopically confirmed peritoneal metastasis outside the pelvis and regional lymph node metastasis.
Stage IV	Distant metastasis involves the liver, lung, or other organs.

Importance of Accurate Staging

Treatment planning: Staging is a critical guide for developing tailored treatment plans. Depending on the stage of the disease, healthcare providers can determine the most appropriate therapeutic modalities for the patient. For example, early-stage disease (Stage I) may be amenable to surgery alone, while advanced-stage disease (Stages III and IV) often necessitates surgery, chemotherapy, and possibly radiation therapy. Accurate staging ensures that treatment decisions are well-informed and optimized, offering the best chance for successful disease management and improved patient outcomes [18].

Prognostic evaluation: Staging is essential for estimating a patient's prognosis. The tumor stage is a critical prognostic factor, as it provides valuable insights into the extent of the disease and its potential spread. Patients with early-stage disease generally have a more favorable prognosis than those with advanced-stage disease. Prognostic assessment informs patients and their healthcare teams about the likely course of the disease, aiding in discussions about treatment goals, potential outcomes, and long-term care planning [19].

Research and clinical trials: Accurate staging is crucial for the enrollment of patients in clinical trials. Clinical trials are instrumental in evaluating the effectiveness of new treatment approaches and emerging therapies for ovarian germ cell tumors. Researchers and healthcare providers rely on the staging system to ensure that patients are appropriately categorized and eligible for specific trials. Participation in clinical trials not only provides patients with access to cutting-edge treatments but also contributes to the advancement of medical knowledge and the development of more effective therapies [20].

Follow-up and monitoring: Staging offers a baseline for monitoring the response to treatment and assessing disease recurrence during follow-up care. It provides a standardized framework for evaluating tumor status changes over time. By regularly assessing the stage and employing imaging and tumor marker monitoring, healthcare providers can track the patient's progress, detect any signs of recurrence or treatment resistance, and make necessary adjustments to the ongoing care plan. This proactive approach is essential for long-term disease management and preservation of the patient's quality of life [21].

Imaging in Staging

CT: CT scans of the abdomen and pelvis are frequently employed to understand the tumor's extent, lymph node involvement, and distant metastases. CT scans use X-ray technology and computer processing to create cross-sectional images of the abdominal and pelvic regions. This imaging modality is especially valuable for identifying ovarian germ cell tumor size, location, and characteristics. It also aids in visualizing the involvement of adjacent structures such as the uterus, bladder, or rectum. Furthermore, CT scans are instrumental in identifying the presence of lymph node enlargement and distant metastases, allowing healthcare providers to accurately stage the disease and plan the most appropriate treatment [22].

MRI: MRI is highly effective in assessing the local tumor's extent and providing detailed information about the tumor's relationship with neighboring structures. It is beneficial for identifying tumor invasion into adjacent structures within the pelvis and abdomen. MRI offers exceptional soft tissue contrast and can help determine whether the tumor has infiltrated structures such as the uterus, bladder, or rectum. Additionally, MRI is employed for assessing the status of pelvic and abdominal lymph nodes. This imaging modality assists in accurate staging and is a vital tool in preoperative evaluation, facilitating surgical planning and determining treatment strategies [23].

PET scans: PET scans may be utilized to detect distant metastases and assess the metabolic activity of the tumor. In PET imaging, active cells, including cancer cells, administer and absorb a small amount of radioactive material. PET scans can identify areas of high metabolic activity, indicating tumor presence or metastasis. This modality is especially valuable for identifying distant metastases in organs outside the pelvic and abdominal regions, helping healthcare providers comprehensively stage the disease and make informed treatment decisions [24].

Chest X-rays: As ovarian germ cell tumors can metastasize to the lungs, chest imaging is often performed to evaluate the presence of lung metastases. Chest X-rays or CT scans provide a means to assess the condition of the lungs and identify any metastatic lesions. Detecting lung metastases is vital for accurate staging and determining the extent of the disease, which, in turn, guides treatment planning and prognostic evaluation [25].

Treatment modalities

Surgery

Fertility-sparing surgery: Fertility-sparing surgery is critical for young women diagnosed with ovarian germ cell tumors who wish to preserve their reproductive potential. This approach involves the removal of the affected ovarian tissue while sparing the unaffected ovary. The extent of fertility-sparing surgery depends on the tumor's stage, histological subtype, and patient's desire for future fertility. In some cases, removing only the tumor (resection or excision) or performing unilateral oophorectomy while leaving the contralateral ovary intact may be possible. This approach aims to balance oncological safety and preserving the patient's ability to conceive [26].

Radical surgery: In cases where fertility-sparing surgery is not feasible or in advanced-stage disease, radical surgery may be necessary. Radical surgery involves a total abdominal hysterectomy, bilateral salpingo-oophorectomy, and the removal of any visible tumor implants within the abdominal cavity. The stage and extent of the disease determine the extent of surgery. Adjuvant treatments such as chemotherapy and, in some cases, radiation therapy may follow radical surgery [27].

Chemotherapy: Chemotherapy is a foundational approach to managing ovarian germ cell tumors. The selection of chemotherapy regimens depends on the tumor's histology and stage. Among the commonly utilized chemotherapy drugs are platinum-based agents such as cisplatin or carboplatin, frequently administered in combination with etoposide or bleomycin. However, tailored chemotherapy regimens may be recommended for specific subtypes, such as yolk sac tumors or choriocarcinomas. The number of treatment cycles and the duration are individualized to align with the patient's clinical response and the stage of the disease. Concurrently, research into emerging chemotherapy regimens and targeted therapies aims to enhance the treatment landscape for ovarian germ cell tumors. Novel agents, evolving treatment protocols, and combination therapies are under investigation through clinical trials to identify more effective and less toxic treatments to bolster patient prognosis [28].

In contrast, though less commonly employed, radiation therapy assumes a role in specific scenarios. It is primarily considered for patients with non-seminomatous tumors that are unresectable or unresponsive to chemotherapy. Radiation therapy may also be a consideration for seminomatous tumors necessitating adjuvant treatment. Modern radiation techniques such as intensity-modulated radiation therapy (IMRT) and stereotactic body radiation therapy (SBRT) are pivotal in minimizing collateral damage to healthy tissues [29].

Another promising avenue lies in targeted therapies marked by an increasing focus on ovarian germ cell tumors. Targeted therapies pinpoint molecular pathways or unique biological markers specific to the tumor, thus heightening effectiveness and mitigating side effects. These therapies may be employed with chemotherapy or as standalone treatments, fostering optimism for improved response rates and long-term outcomes [30].

Additionally, immunotherapy, including immune checkpoint inhibitors, represents an innovative approach in the treatment landscape. By modulating the immune system's response, these therapies can potentially manage a spectrum of malignancies, including gynecological cancers. The integration of immunotherapy into a comprehensive treatment strategy is an avenue under exploration, with ongoing research dedicated to assessing its efficacy in the context of ovarian germ cell tumors. These multifaceted treatment options underscore the commitment to improving the care and prognosis of patients facing this rare and complex group of tumors [31].

Prognosis and predictive factors

Factors Influencing Prognosis

Histological subtype: The specific histological subtype of the tumor significantly affects prognosis. For example, patients with dysgerminomas tend to have a more favorable prognosis than those with yolk sac tumors or choriocarcinomas. Each subtype has its own clinical behavior and response to treatment, which ultimately influences patient outcomes. Understanding the histological subtype is essential for tailoring treatment plans and predicting prognosis accurately [32].

Stage: The stage of the tumor at the time of diagnosis is a critical determinant of prognosis. Ovarian germ cell tumors are typically staged using the FIGO staging system. Patients with early-stage disease (Stage I) generally have a more favorable prognosis than those with advanced-stage disease (Stages III and IV). Early diagnosis and intervention are crucial in improving outcomes, as early-stage tumors are more amenable to curative treatment [33].

Age: The patient's age at diagnosis is associated with prognosis. Adolescents and young adults tend to have more favorable outcomes than older individuals. This age-related difference in prognosis may be due to various factors, including the younger patients' overall health and physiological resilience [34].

Completeness of surgery: The success of surgical intervention, particularly radical surgery aimed at removing all visible tumors (complete cytoreduction), significantly influences prognosis. Achieving complete cytoreduction is associated with improved survival rates, as it reduces the tumor burden and enhances the effectiveness of subsequent treatments such as chemotherapy [35].

Response to chemotherapy: The patient's response to chemotherapy is a critical prognostic factor. Patients who respond well to chemotherapy and experience tumor shrinkage typically have better outcomes. The response assessment often involves radiological imaging and monitoring tumor markers. The effectiveness of chemotherapy in reducing the size and activity of the tumor can help predict the patient's prognosis [36].

Tumor markers: Tumor markers, such as AFP and β-hCG, are important prognostic indicators. Elevated marker levels at diagnosis may be associated with a poorer prognosis, as they often reflect the extent of the disease. Conversely, a decline in marker levels during treatment is a positive sign and suggests a better response to therapy [37].

Recurrence: If it occurs, the timing and pattern of disease recurrence can impact prognosis. Early recurrences, especially during or shortly after treatment, may be more challenging to manage and associated with a worse prognosis. Whether localized or distant, the site of recurrence also plays a role in prognosis. Surveillance and regular follow-up are crucial for the early detection and management of recurrent disease [38].

Predictive Biomarkers

AFP and β-hCG levels: AFP and β-hCG levels serve as diagnostic tools and predictive biomarkers. Monitoring changes in AFP and β-hCG levels during treatment is crucial for assessing the response to therapy. A decrease in these marker levels during chemotherapy is typically associated with a positive response, while rising or persistent elevated levels may indicate resistance or disease progression. Continually monitoring these markers allows healthcare providers to make real-time treatment adjustments, optimizing the patient's chances of a successful outcome [39].

Molecular markers: Emerging research in ovarian germ cell tumors is identifying molecular markers that can guide treatment decisions. For example, specific genetic mutations or biomarkers associated with certain subtypes of these tumors may influence the choice of targeted therapies. Molecular profiling offers valuable insights into the distinct genetic characteristics of ovarian germ cell tumors, potentially unveiling vulnerabilities that can be targeted with specific treatments. Understanding the molecular underpinnings of ovarian germ cell tumors allows for more precise and personalized treatment strategies, improving the chances of a successful response to therapy and a better prognosis [40].

Expression of immune markers: In the era of immunotherapy, the expression of immune markers on the tumor cells and within the tumor microenvironment is of great interest. These markers can offer insights into the tumor's interactions with the immune system and may predict the response to immunotherapies. Immune checkpoint inhibitors and other immunotherapeutic approaches are studied to treat ovarian germ cell tumors. By examining the expression of immune markers, healthcare providers can better identify patients who will likely respond favorably to immunotherapy, allowing for more tailored and effective treatment plans [41].

Long-Term Outcomes

Early-stage disease (Stage I): Patients diagnosed with early-stage disease (Stage I) typically experience excellent long-term survival rates. Many of these individuals achieve complete remission, leading to long-term disease-free status. The relative tumor containment within the ovaries in Stage I contributes to a favorable prognosis. Treatment measures such as surgery and adjuvant therapy can effectively eliminate the disease [42].

Advanced-stage disease (Stages III and IV): Long-term outcomes for patients with advanced-stage disease (Stages III and IV) can be more challenging. The extent of disease involvement beyond the ovaries can complicate treatment and prognosis. However, aggressive and comprehensive treatment approaches, including surgery and chemotherapy, can still yield favorable outcomes for some patients. Advances in treatment options, including targeted therapies and immunotherapies, further improve long-term outcomes for those with advanced disease [42].

Fertility-sparing surgery: The availability of fertility-sparing surgery is a notable advancement in ovarian germ cell tumors. This approach allows many young women to preserve their reproductive potential while achieving favorable long-term survival. For patients who desire to retain their fertility, this option offers a crucial balance between treating the disease and safeguarding their future family planning [42].

Regular follow-up and monitoring: Long-term outcomes are greatly influenced by ongoing follow-up and monitoring. Post-treatment surveillance is essential for assessing the patient's long-term progress and detecting potential disease recurrences. Regular check-ups, imaging studies, and tumor marker assessments are integral to post-treatment care. Timely detection of recurrences allows for prompt intervention and maximizes the chances of successful disease management [42].

Challenges and limitations

Late Diagnosis

Nonspecific symptoms: Ovarian germ cell tumors often manifest with nonspecific symptoms, such as abdominal pain and swelling. These vague symptoms can easily be attributed to other common conditions, including gastrointestinal issues or menstrual discomfort. As a result, patients may not immediately recognize these symptoms as potentially indicative of a severe medical condition. The lack of specificity in tumor presentation can lead to delays in the diagnosis and the initiation of appropriate treatment, hindering the patient's chances of early intervention and a better prognosis. Increasing awareness among patients and healthcare providers about these symptoms is essential for timely evaluation and diagnosis [7].

Misdiagnosis: Given the rarity of ovarian germ cell tumors, healthcare providers may not always have them at the forefront of their diagnostic considerations. Consequently, these tumors can be misdiagnosed as other more common gynecological conditions or ovarian cancers. Misdiagnosis can result in inappropriate treatments and delays in receiving the correct diagnosis and the most effective care. Ensuring that healthcare providers are informed about the existence and characteristics of ovarian germ cell tumors is crucial in reducing the rate of misdiagnosis and ensuring that patients receive the most appropriate care from the outset [43].

Lack of awareness: The rarity of ovarian germ cell tumors may contribute to a lack of awareness among healthcare providers and the general public. The limited awareness about these tumors can impede their early detection and diagnosis. Raising awareness is paramount in facilitating early detection and intervention. Education efforts should target healthcare professionals, patients, and the community, helping them recognize the signs and symptoms associated with ovarian germ cell tumors. Such awareness campaigns can improve outcomes by ensuring that these tumors are considered in the differential diagnosis and that patients receive timely evaluation and appropriate care [44].

Treatment-Related Side Effects

Chemotherapy side effects: Chemotherapy, a cornerstone of treatment for ovarian germ cell tumors, can elicit various side effects that affect a patient's quality of life. Nausea, vomiting, fatigue, hair loss, and hematological issues such as anemia, neutropenia, and thrombocytopenia are common chemotherapy-related side effects. Patients often require close monitoring and supportive care to manage these adverse effects. Antiemetic medications help control nausea and vomiting, while blood transfusions, growth factors, and medications may address hematological issues. Oncology nurses and supportive care teams are pivotal in providing guidance and interventions to alleviate chemotherapy side effects, enhancing the patient's well-being and treatment adherence [45].

Fertility issues: Preserving fertility is a significant concern, particularly for young women diagnosed with ovarian germ cell tumors. While fertility-sparing surgery is an option for some patients, others may require radical surgery or aggressive chemotherapy regimens that can impact fertility. This underscores the importance of discussing fertility preservation options early in the treatment planning. For those who desire future family planning, fertility preservation methods such as egg or embryo freezing can provide hope and options for preserving the ability to conceive after completing cancer treatment. Providing information and emotional support around fertility decisions is essential, acknowledging the profound impact of cancer treatment on reproductive concerns [46].

Long-term health concerns: Survivors of ovarian germ cell tumors may face enduring health concerns beyond their initial treatment. These concerns can encompass the risk of secondary malignancies, cardiovascular issues, and endocrine complications. Ovarian germ cell tumors and their treatments can affect long-term health, highlighting the importance of regular follow-up and survivorship care. Health professionals specialized in survivorship care can provide ongoing monitoring, manage the risk of late effects, and address concerns about the patient's overall well-being. This comprehensive care approach ensures that survivors receive the necessary support and interventions to mitigate the cancer's potential long-term health consequences and its treatment [47].

Resistance to Therapy

Chemoresistance: Chemoresistance is a formidable challenge in treating ovarian germ cell tumors. Over time, some tumors may resist the chemotherapy drugs used in their initial treatment, rendering these agents less effective or entirely ineffective. The emergence of chemoresistance can lead to treatment failure and disease progression. Identifying and overcoming chemoresistance is a complex and ongoing endeavor in oncology. Research focuses on understanding the mechanisms underlying resistance and developing strategies to counteract it, such as combination therapies or novel agents to restore drug sensitivity [48].

Limited treatment options: The limited number of effective treatment options, particularly for refractory or recurrent ovarian germ cell tumors, presents a significant challenge. After initial treatment, few standard therapies may be available for managing disease recurrence, and these may need more efficacy. This limitation underscores the urgent need for ongoing research into innovative treatment modalities, including targeted therapies and immunotherapies. Developing new, more effective treatments is critical in providing hope and improved outcomes for patients facing disease relapse [49].

Tumor heterogeneity: Ovarian germ cell tumors exhibit histological and molecular heterogeneity. This diversity in tumor characteristics makes predicting which treatments will be most effective for individual patients challenging. Treatment decisions are often based on the tumor's histological subtype and stage. Still, as our understanding of the molecular underpinnings of these tumors evolves, more personalized treatment approaches may become possible. Precision medicine, guided by molecular profiling and the identification of relevant biomarkers, holds promise in tailoring treatment strategies to individual patients, addressing the issue of tumor heterogeneity [50].

Relapse and recurrence: Despite successful initial treatment, relapse and recurrence of ovarian germ cell tumors can occur. Managing recurrent diseases can be complex and may require innovative approaches. Patients facing relapse may benefit from additional surgeries, alternative chemotherapy regimens, targeted therapies, or participation in clinical trials investigating novel treatments. Timely and thorough monitoring is essential for the early detection of recurrence, enabling the prompt initiation of appropriate interventions. Managing relapse and recurrent disease is an ongoing aspect of care for patients with ovarian germ cell tumors, necessitating close collaboration between healthcare providers and patients [51].

Supportive care

Symptom Management

Pain management: Pain, particularly in the abdominal or pelvic region, is a common symptom associated with ovarian germ cell tumors. Effective pain management is essential to enhance the patient's quality of life. It involves a combination of pharmacological and non-pharmacological approaches. Medications, including analgesics and opioids, can be prescribed to alleviate pain. Non-pharmacological strategies such as relaxation techniques, heat therapy, and physical therapy may also complement pain relief and improve the patient's overall comfort [52].

Nausea and vomiting control: Chemotherapy-related nausea and vomiting are distressing side effects that can significantly impact a patient's well-being. Various antiemetics medications are administered to prevent and alleviate these symptoms. Antiemetic therapies are tailored to the specific chemotherapy regimen used. Ensuring effective control of nausea and vomiting is vital in promoting patient compliance with chemotherapy and reducing treatment-related distress [53].

Fatigue management: Cancer-related fatigue is a pervasive and debilitating concern for many patients undergoing treatment for ovarian germ cell tumors. Fatigue can affect a patient's daily functioning, mood, and overall quality of life. Management strategies encompass a holistic approach. Exercise programs tailored to the patient's physical capabilities can help combat fatigue and improve energy levels. Lifestyle modifications, including a consistent sleep schedule and a balanced nutrition, contribute to energy preservation. Psychological support and counseling play a crucial role in addressing the emotional aspects of fatigue, providing patients with coping strategies to navigate this challenging symptom [54].

Nutritional support: Maintaining adequate nutrition is vital during cancer treatment. Ovarian germ cell tumors and their treatments can affect appetite and digestion, making it challenging for patients to obtain essential nutrients. Nutritionists and dietitians are integral in guiding patients on dietary choices that meet their nutritional needs and mitigate the side effects that may affect their eating ability. Nutritional support aims to ensure that patients receive adequate sustenance to support their physical strength and immune system and address any weight changes and dietary concerns that may arise during treatment [55].

Hematological support: Patients undergoing chemotherapy for ovarian germ cell tumors may experience various blood-related side effects, including anemia, neutropenia, and thrombocytopenia. Managing these hematological issues is paramount for the patient's safety and well-being. Supportive care measures may include blood transfusions to address anemia, medications to stimulate white blood cell production in the case of neutropenia, and platelet transfusions or medications to manage thrombocytopenia. These interventions aim to prevent complications, such as infections and bleeding, which can arise from compromised blood counts [56].

Fertility preservation: For young women diagnosed with ovarian germ cell tumors who wish to preserve their fertility, supportive care encompasses counseling on fertility preservation options. This may include discussions about egg or embryo freezing, which can allow patients to pursue family planning in the future. Fertility preservation is a significant aspect of supportive care that acknowledges the patient's emotional and reproductive concerns, providing them with the information and resources they need to make informed decisions about their future family-building goals [57].

Psychological and Emotional Support

Counseling and psychotherapy: Coping with a cancer diagnosis can be emotionally taxing. Patients often grapple with anxiety, depression, and stress, significantly impacting their well-being. Individual or group counseling, facilitated by mental health professionals, offers a safe and confidential space for patients to express their emotions, fears, and concerns. Psychotherapeutic approaches, such as cognitive-behavioral therapy (CBT), are valuable tools in helping patients develop coping strategies, challenge negative thought patterns, and manage the emotional burden accompanying cancer. CBT, for instance, can assist individuals in reframing their thoughts and developing more positive and adaptive responses to their challenges [58].

Support groups: Participation in support groups gives patients a sense of community and emotional support. Connecting with others who have experienced similar diagnoses and treatments can be profoundly reassuring and empowering. Support groups offer an avenue for sharing experiences, exchanging advice, and finding solace in the company of those who understand the unique challenges of facing a cancer diagnosis. These groups provide emotional support and foster a sense of belonging and unity among patients, helping to reduce feelings of isolation [59].

Patient and family education: A well-informed patient is better equipped to make decisions about their treatment and care. It is essential to provide patients and their families of comprehensive information about the disease, treatment options, and potential side effects. Education empowers individuals to participate in their healthcare decisions actively, alleviates anxiety arising from uncertainty, and facilitates effective communication with the healthcare team. Moreover, when families are educated about the disease and its management, they can offer their loved ones more meaningful and informed support [60].

Spiritual and religious support: Faith and spirituality are integral to many patients' lives. In times of illness and emotional distress, spiritual or religious support can provide solace, meaning, and a sense of connection to a higher power or community. Chaplains and spiritual counselors are available to help patients navigate their cancer journey's spiritual and emotional aspects, offering comfort, guidance, and a space for prayer or reflection in alignment with their faith [61].

Palliative care: Palliative care is a holistic approach to managing pain, symptoms, and emotional distress throughout the cancer journey. Palliative care teams, including palliative care physicians and specialized nurses, focus on enhancing patients' quality of life. They relieve pain and discomfort, offer emotional support, and address spiritual and existential concerns. Importantly, palliative care is not limited to end-of-life care; it can be integrated at any stage of treatment. Palliative care complements curative treatments, ensuring patients receive the most comprehensive and compassionate care [62].

Survivorship care: As patients transition into survivorship, they may face challenges beyond treatment. The fear of cancer recurrence, body image issues, and concerns about long-term health are common aspects of survivorship. Ongoing psychological and emotional support is essential during this phase. Healthcare providers and survivorship care teams offer guidance, regular follow-up, and resources to help patients navigate the emotional complexities of survivorship. This support is vital to help survivors regain a sense of normalcy, rebuild their lives, and address any lingering emotional distress [63].

## Conclusions

This comprehensive review has shed light on the intricate landscape of ovarian germ cell tumors, providing insights into their diverse classification, epidemiology, diagnostic methods, staging, and evolving treatment modalities. Current trends in diagnosis and treatment emphasize the importance of personalized care based on the tumor's histological subtypes, stage, and patient's age. Fertility-sparing surgery, chemotherapy, targeted therapies, and emerging treatment options offer promise for enhanced outcomes. At the same time, advanced imaging techniques are pivotal in precise staging and tailored treatment planning. Given the challenges of nonspecific symptoms and misdiagnosis, early detection remains a fundamental goal, underscoring the need for heightened awareness among healthcare providers and the public.

Multidisciplinary care is the cornerstone of effective management, with gynecologic oncologists, medical oncologists, radiologists, pathologists, and supportive care teams collaborating to create individualized treatment plans that maximize patient outcomes. Preserving fertility, particularly for young women, is critical, and comprehensive support is imperative. The future of ovarian germ cell tumor management is promising, with ongoing research and evolving clinical practice opening new avenues for improving outcomes and reducing side effects. Identifying predictive biomarkers, addressing chemoresistance, and enhancing awareness are critical focus areas, offering hope for a brighter future for individuals diagnosed with these rare tumors. As knowledge and expertise in this field expand, patients and their families can expect more effective treatments and improved quality of life.
